# A New Species of the Genus *Scybalocanthon* (Coleoptera: Scarabaeinae) from Southeast Brazil

**DOI:** 10.1371/journal.pone.0027790

**Published:** 2011-11-14

**Authors:** Fernando Augusto Barbosa Silva

**Affiliations:** 1 Universidade Federal de Lavras, Departamento de Entomologia, Campus Universitário, Lavras, Brazil; 2 Universidade Federal de Lavras, Departamento de Biologia, Setor de Ecologia, Campus Universitário, Lavras, Brazil; University of Guelph, Canada

## Abstract

A new species of the genus *Scybalocanthon* Martínez 1948, *Scybalocanthon korasakiae* **sp. nov.**, from Atlantic forest ecosystem, state of Minas Gerais, Brazil is described based on external and genital morphology.

## Introduction

The genus *Scybalocanthon* Martínez (1948) is a small genus of dung beetle from Central and South America [1]. In the original description of the genus, Martínez included only one species, *Canthon moniliatus* Bates 1887, type species of the genus by monotypy [2]. In 1949, Martínez revised his original description adding the following characters: pygidium lacking basal margin separating it from the propygidium and proepisternum lacking denticle or carina across the lateral margim [3].

Until 1956, the genus had only three described species: *Scybalocanthon moniliatus*, *S. zischkai* and *S. darlingtoni* [1]. The first major taxonomic work on the genus was Pereira and Martínez in 1956, who described a new species (*S. kelleri*) and transferred six species from *Canthon* Hoffmannsegg, 1817 [1]. In 1972, Martínez and Halffter described another species (*S. balachowskyi*) [4]. In 2010, Molano and Medina described *S. arcabuquensis* and added introduced consideration of male genitalic characters [5]. All other species that are now placed in the genus *Scybalocanthon* were transferred from *Canthon*, which appears to be very close phylogenetically [6].

Heretofore the genus has comprised 17 described species: *S. aereus* (Schmidt 1922); *S. arcabuquensis* Molano & Medina 2010; *S. balachowskyi* Martínez & Halffter 1972; *S. cyanocephalus* (Harold 1868); *S. darlingtoni* (Paulian 1939); *S. imitans* (Harold 1868); *S. kastneri* (Balthasar 1939); *S. kelleri* Pereira & Martínez 1956; *S. maculatus* (Schmidt 1920); *S. moniliatus* (Bates 1887); *S. nigellus* (Schmidt 1922); *S. nigriceps* (Harold 1868); *S. pinopterus* (Kirsch 1873); *S. pygidialis* (Schmidt 1922); *S. sexspilotum* (Guérin-Méneville 1855); *S. trimaculatus* (Schmidt 1922) and *S. zischkai* Martínez 1949 [5]. In this work a species of *Scybalocanthon* from the Atlantic forest ecosystem in Minas Gerais state, southeastern Brazil is described.

The main diagnostic characteristics that separate the *Scybalocanthon* species from related species in the genus *Canthon* are a) basal tarsomere of mesotarsus and metatarsus short, length about one-half that of second tarsomere, and obliquely truncated apically; b) lateral borders of tarsomeres parallel, forming an even margin along the length of the tarsus; c) overall shape of tarsomeres 2–4 quadrate to rectangular [2], [7].

## Materials and Methods

Beetles were collected under collecting permit number 10061-1 SISBIO/IBAMA issued by the Universidade Federal de Lavras for permanent researchers. The collecting locations reported in this work were not protected in any way and the field studies did not involve endangered or protected species.

The 74 adult specimens comprising the type series were collected in an Atlantic Forest fragment in the city of Lavras, southern Minas Gerais state, Brazil. The sample area consists of a transition region between Cerrado and Atlantic Forest.

The specimens studied have been deposited in the following plubic collections: MZUSP – Museu de Zoologia da Universidade de São Paulo; MZUEFS – Museu de Zoologia da Universidade Estadual de Feira de Santana; MZUFMT – Museu de Zoologia da Universidade Federal de Mato Grosso; CRENUFLA – Coleção de Referência de Escarabeíneos Neotropicais, Laboratório de Ecologia de Invertebrados Terrestres, Universidade Federal de Lavras.

The holotype photos were captured using a Leica stereomicroscope M205A coupled to an Automontage system (Leica Application Suite, version 3.7.0). The internal sclerites were removed from aedeagus and studied in the same position in which they were observed. The nomenclature used to describe the structures of the male genital organ is that proposed by Molano & Medina [5].

### Nomenclatural Acts

The electronic version of this document does not represent a published work according to the International Code of Zoological Nomenclature (ICZN), and hence the nomenclatural acts contained in the electronic version are not available under that Code from the electronic edition. Therefore, a separate edition of this document was produced by a method that assures numerous identical and durable copies, and those copies were simultaneously obtainable (from the publication date noted on the first page of this article) for the purpose of providing a public and permanent scientific record, in accordance with Article 8.1 of the Code. The separate print-only edition is available on request from PLoS by sending a request to PLoS ONE, 1160 Battery Street, Suite 100, San Francisco, CA 94111, USA along with a check for $10 (to cover printing and postage) payable to “Public Library of Science”.

In addition, this published work and the nomenclatural acts it contains have been registered in ZooBank, the proposed online registration system for the ICZN. The ZooBank LSIDs (Life Science Identifiers) can be resolved and the associated information viewed through any standard web browser by appending the LSID to the prefix “http://zoobank.org/”. The LSID for this publication is: (urn:lsid:zoobank.org:pub:802F7A89-8A3D-4770-B17E-115B7763E6C0).

## Results and Discussion

### Taxonomic treatment

***Scybalocanthon korasakiae*** **sp. nov.** (urn:lsid:zoobank.org:act:E2BFD82D-208B-4547-BBDE-7F9D6EB1AAD2).

### Etymology

This species is named in honour of Vaneska Korasaki, a specialist in Amazonian conservation and dung beetle ecology.

### Diagnosis

**Figure 1:**
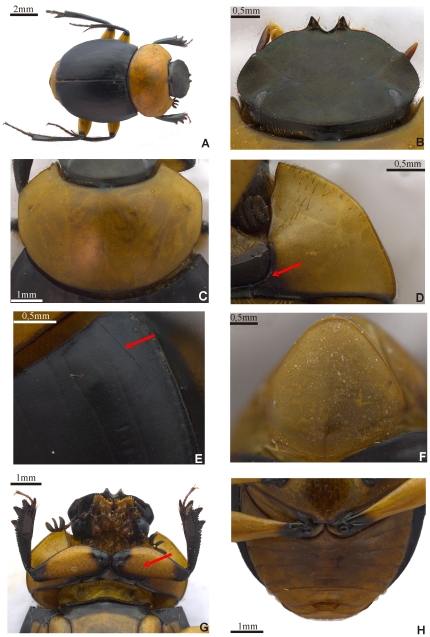
*Scybalocanthon korasakiae* sp. nov. (A) holotype MZUSP, (B) head, (C) pronotum, (D) hypomeron with keel on internal border (arrow points to the keel), (E) eighth elytral stria carinated (arrow points to the keel), (F) pygidium, (G) ventral view of the anterior part of body (arrow points to multiple rows of setae covering the middle region of profemur along the proximal-distal axis), (H) male abdominal ventrites.

**Figure 2:**
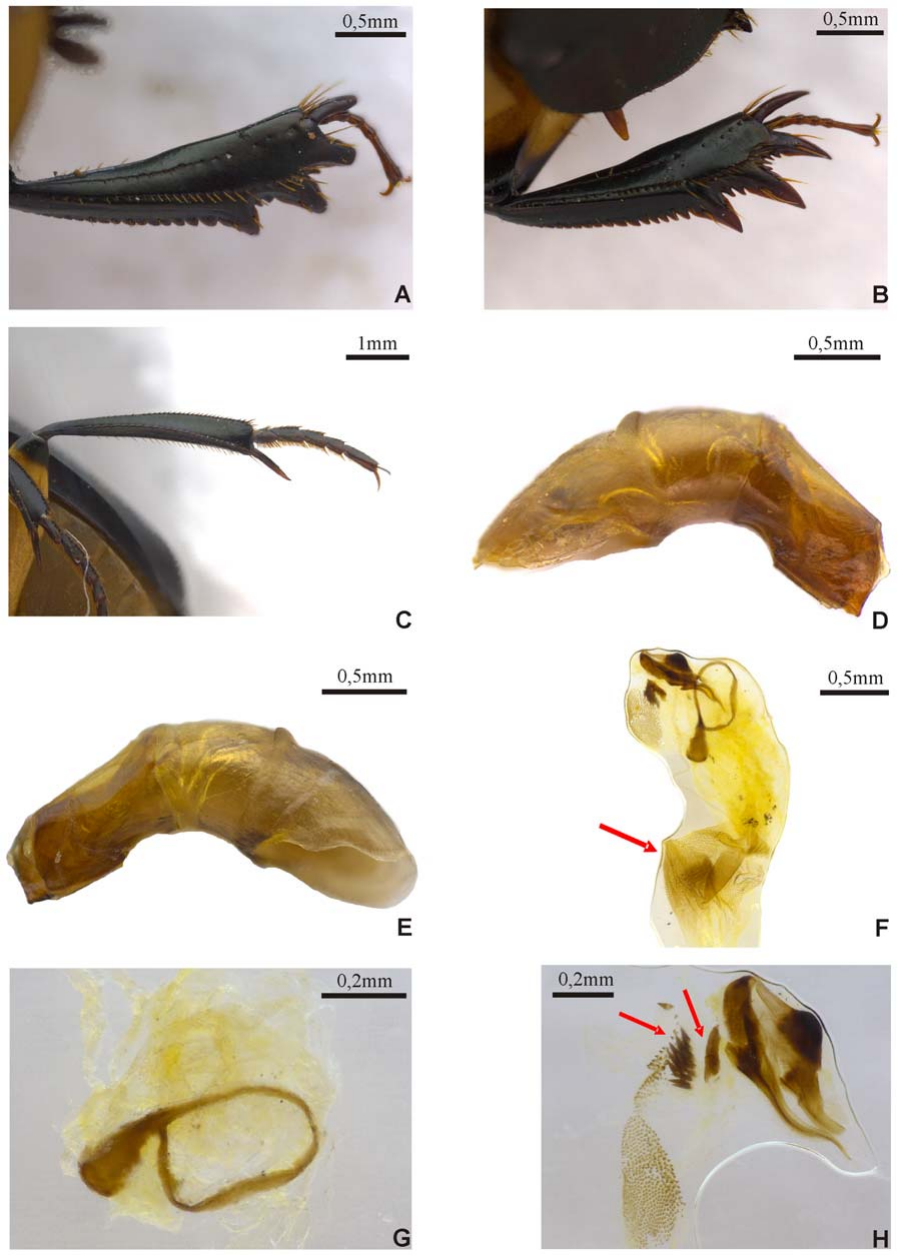
*Scybalocanthon korasakiae* sp. nov. (A) male protibiae, (B) female protibiae, (C) male metatibiae, (D) aedeagus (right side), (E) aedeagus (left side), (F) internal sac of aedeagus (arrow points to the lobe), (G) basal sclerite, (H) elongated sclerite (upper right), plate sclerite (lower left) and attached to elongated and plate sclerites there are two loose sclerites at inner edge indicated by arrows.

The type series displays a unique color combination (head, pronotum, elytra, pygidium and thoracic appendages) for *Scybalocanthon* (Figures 1A, F, G and H). Posterior angle of pronotum with a small hollow. Hypomeron with smooth keel on internal border (Figure 1D). Elytral striae impressed, with poorly defined punctures. Eighth stria with thin carina at the base (Figure 1E); the striae disappears posterior to the carina and reappears in the middle of elytron. Interstriae with very small, inconspicuous, setigerous punctures. Abdominal ventrites with numerous small setae the same color as abdomen (brownish-yellow) (Figure 1H). Pygidium with numerous, randomly distributed yellowish-brown setae (Figure 1F). Profemur with strip of setae covering middle region along the proximal-distal axis (Figure 1G). Surfaces of meso- and metafemora glabrous. Shape of parameres and structures of the internal sac are also unique within the genus (Figures 2D–H).

### Description (Holotype)

Body. Oval, lateral edges rounded (Figure 1A). Colour. Head, anterior and posterior edge of pronotum, elytra, prosternum, internal edge of hypomera, mesosternum, mesoepisternae, procoxae, trochanters, tibiae, meso and metatarsomeres, basal and apical parts of femurs black with greenish or bluish reflection; other parts of the pronotum, pygidium, most of the hypomera, metasternum, metaepistenum, abdomen, most of the middle and posterior coxae, and femora (except the extremities) yellowish-brown color. Length, 10 mm. Body size of specimens examined varies from 8 to 10.7 mm. Head. Dorsal surface covered with fine punctures, difficult to see under low magnification. Lateral edge bordered by a row of small setae. Clypeal border with two small, triangular central teeth, somewhat sharp, separated by a V-shaped notch (Figure 1B). Anterior edge of the head following a smooth arc from clypeal teeth to clypeo-genal suture. Behind clypeo-genal suture, the edge has a small angle before following a smooth arc to the eye. Clypeo-genal suture fine and inconspicuous. Clypeo-frontal suture brighter than adjacent cuticle. Dorsal interocular width 14 times width eye. Posterior dorsal border of head (occipital region of front) completely margined by fine carina (Figure 1B). Thorax. Pronotum twice as wide as long; moderately convex (Figure 1C), anterior angles acute, directed forward; finely punctate and glabrous, punctures noticeable only at high magnification. Lateral pronotal impressions with small black spot poorly differentiated from adjacent surface. Posterior pronotal angle with small hollow. Hypomeron not excavated, with smooth keel on internal border and without tubercle on lateral border (Figure 1D); lacking carina separating the anterior and posterior area; anterior surface and lateral border with small, sparse setae. Mesosternum slightly rugose. Metasternum gently convex and smooth. Elytra. Nine well-defined, grooved striae with weak punctures. Eighth striae with a thin carina at the base (Figure 1E) that disappears after the carina and reappears in the middle of elytron. Interstriae with very small, setigerous punctures, barely visible; setae visible only at high magnification. Width of sixth interstria at base about one-half width at midway point. Abdomen. Ventrites with numerous small setae the same color as abdomen (brownish-yellow) (Figure 1H); setae are difficult to view due to lack of contrast with abdomen. Lateral region of ventrites 3, 4, 5, and 6 with a higher concentration of setae. Central region of the fourth and sixth ventrites with length greater than others ventrites. Pygidium rounded apically, clearly margined, with numerous randomly distributed yellowish-brown setae (Figure 1F). Pygidium not separated from propygidium by carina. Thoracic appendages. Profemur with multiple row of setae covering the middle region along the proximal-distal axis, and on anterior and posterior edges (Figure 1G). Protibiae with three lateral teeth, apical two closer together than basal two (Figure 2A). In females, apex of the medial tibial tooth is about halfway between the apical and basal tooth (Figure 2B). Protibial spur subcylindric, with a slight curvature at the base and apex truncated. Surfaces of meso- and metafemora glabrous. Anterior and posterior borders of meso- and metafemur not margined. First meso and metatarsomeres short, about half the length of second tarsomere, obliquely truncated at apex (Figure 2C). Tarsal claws without basal teeth. Male genital organ. Subrectangular and asymmetrical parameres (Figures 2D, E). Internal sac with submedial lobe and small brush-shaped teeth (Figure 2F). Medial sclerites absent. Basal sclerite circular, with semirecto handle-shaped extension and ring with regular border (Figure 2G). Plate sclerite membranous with elliptical shape and rounded border (Figure 2H). Elongated sclerite irregularly shaped with filamentous projection towards lower border (Figure 2H). Attached to elongated and plate sclerites are two loose sclerites at inner edge, one tooth-shaped and other asymmetric (Figure 2H). Secondary sexual characteristics.

The above description was based on the male holotype. Females differ from males in the following characters: middle protibial tooth equidistant from the other teeth (Figure 2B); apex of tibial teeth and tibial spur more pointed than that of males (Figure 2B); sixth abdominal ventrite longer than in males.

### Type material

**Holotype, BRAZIL:** MINAS GERAIS, Lavras, (21°16.615′S, 44°57.072′W), 950 m, 21.I.2011, C. M. Q. Costa & R. Maciel – 1 ♂ (MZUSP); **Allotype**, same data collection of the holotype – 1 ♀ (MZUSP); **Other Paratypes**, same data collection of the holotype – 5 specimens (MZUEFS – from 53393 to 53397); 12 specimens (MZUSP); 25 specimens (MZUFMT); 30 specimens (CRENUFLA).

### Biological Aspects

According to Molano & Medina [5], the species of the genus *Scybalocanthon* are widely distributed in low wet and dry forests throughout Central and South America. They are usually diurnal, especially during peaks of solar radiation [4]. All specimens studied here were collected in forest fragments with pitfall traps baited with human excrement. This habitat preference and feeding habit are the most common among the species of *Scybalocanthon* for which biological data are available [4], [5].

### Remarks

Specimens examined exhibit no significant variations in external morphology. The same nomenclature for the internal sac sclerites proposed by Molano & Medina [5] was used for comparative issues. However, the plate sclerite of this species, as well as that of some species studied by previous authors, does not present a typical sclerotized structure but is very membranous. Thus, the primary homology of this structure with the plate sclerite of other Deltochilini must be carefully analyzed.

The asymmetry between the parameres of this species is similar to that in other species of the genus treated by Molano & Medina [5]. Thus, although in *S. aureus* the parameres are not be asymmetrical, parameral asymmetry seems to be an important evolutionary character of the genus.

From aspects of the external morphology (eighth striae with a thin carina at the base; coloration of head, pronotum and pygidium) and the structure of the aedeagus and internal sac sclerites, this species is closely related to *S. zischkai*. One of the most striking structures shared between these species and some species cited as “*affines*” to *S. pygidialis*, *S. trimaculatus* and *S. nigriceps* in Molano & Medina [5] is the membranous aspect of the plate sclerite.

An identification key for the species of the genus *Scybalocanthon* was not included because the genus needs taxonomical revision and many of these species can not be securely identified at this moment. Some of the genus species were described in the nineteenth century and these descriptions are superficial. This situation makes the safe identification of some species difficult. Besides, specimens identified in the literature as “*affines*” to valid species should be checked too.

However, the description of this new species has enough information to allow it to be safely identified. The diagnosis, details of morphology of male genitalia and high quality figures presented here improve the identification of specimens. Preliminary studies (unpublished data) noted that this species is restricted to areas of Atlantic Forest. Thus, the correct identification of these specimens may be very useful to conservation projects related to remnants of Atlantic Forest.

The South American Atlantic Forest has important endemic species of dung beetle. However, less than 12 percent of the forest remains and all species that depend on this ecosystem suffer some kind of threat [8]. Thus, biodiversity studies of poorly known groups in this ecosystem are extremely important to science.
